# Vaginal Microflora Associated With Bacterial Vaginosis in Nonpregnant Women: Reliability of Sialidase Detection

**DOI:** 10.1155/S1064744901000047

**Published:** 2001

**Authors:** Jorgelina Smayevsky, Liliana Fernández Canigia, Alejandra Lanza, Hebe Bianchini

**Affiliations:** 1 Laboratorio de Microbiología Centro de Educación Médica e Investigaciones Clínicas Dr. Norberto Quirno CEMIC Buenos Aires Argentina; 2 Camargo 581 Piso 4 Buenos Aires 1414 Argentina

## Abstract

*Objective:* To determine the prevalence of *Gardnerella vaginalis*, anaerobic bacteria and *Mycoplasma hominis* in vaginal specimens of women with and without bacterial vaginosis (BV) as well as to determine the sensitivity and specificity of the direct sialidase assay of vaginal fluid as a rapid test for diagnosing this syndrome.

*Methods:* Vaginal cultures were obtained from 109 nonpregnant women (mean age 33 ± 7.1 years), 47 of them with clinical signs of BV (BV+) and 62 of them without BV (BV- ). In addition, we determined the vaginal sialidase activity in both groups, which may serve as a feature of this syndrome.

*Results:* Anaerobic bacteria were isolated in 91% and 18% of the BV+and BV- groups, respectively (*p* < 0.001). *Peptostreptococcus* spp., *Prevotella bivia* and *Porphyromonas* spp. were strongly associated with BV. *P. bivia* and *Prevotella* spp. represented 44% of all the anaerobes isolated in the BV+ group. All the isolated *P. bivia* strains presented sialidase activity. *G. vaginalis* and *M. hominis* were isolated in 76% and 42% of the BV+ and 1% and 0% of the BV- women, respectively (*p* < 0.001). *Mobiluncus* morphotypeswere observed in 34% of the BV+and 0% of BV- women. Sensitivity, specificity, positive predictive value and negative predictive value of sialidase activity were 81%, 94%, 90% and 86%, respectively.

*Conclusions:* Our data demonstrate a strong association between *G. vaginalis*, *M. hominis*, and *P. bivia* and BV. Sialidase activity and Gram stain of vaginal fluid represent accurate methods for diagnosing BV.


*Infect Dis Obstet Gynecol 2001;9:17–22*

### Vaginal microflora associated with bacterial vaginosis in nonpregnant women: reliability of sialidase detection

**Jorgelina Smayevsky, Liliana Fernández Canigia, Alejandra Lanza and Hebe Bianchini**

*Laboratorio de Microbiología, Centro de Educación Médica e Investigaciones Clínicas Dr. Norberto Quirno CEMIC, Buenos Aires, Argentina*

***Objective:*** To determine the prevalence of *Gardnerella vaginalis,* anaerobic bacteria and *Mycoplasma hominis* in vaginal specimens of women with and without bacterial vaginosis (BV) as well as to determine the sensitivity and specificity of the direct sialidase assay of vaginal fluid as a rapid test for diagnosing this syndrome.
***Methods:*** Vaginal cultures were obtained from 109 nonpregnant women (mean age 33 ± 7.1 years), 47 of them with clinical signs of BV (BV+) and 62 of them without BV (BV–). In addition, we determined the vaginal sialidase activity in both groups, which may serve as a feature of this syndrome.
***Results:*** Anaerobic bacteria were isolated in 91% and 18% of the BV+ and BV– groups, respectively ($p < 0.001$). *Peptostreptococcus* spp., *Prevotella bivia* and *Porphyromonas* spp. were strongly associated with BV. *P. bivia* and *Prevotella* spp. represented 44% of all the anaerobes isolated in the BV+ group. All the isolated *P. bivia* strains presented sialidase activity. *G. vaginalis* and *M. hominis* were isolated in 76% and 42% of the BV+ and 1% and 0% of the BV– women, respectively ($p < 0.001$). *Mobiluncus* morphotypes were observed in 34% of the BV+ and 0% of BV– women. Sensitivity, specificity, positive predictive value and negative predictive value of sialidase activity were 81%, 94%, 90% and 86%, respectively.
***Conclusions:*** Our data demonstrate a strong association between *G. vaginalis*, *M. hominis*, and *P. bivia* and BV. Sialidase activity and Gram stain of vaginal fluid represent accurate methods for diagnosing BV.

Key words: VAGINOSIS; ANAEROBES; DIAGNOSIS

Bacterial vaginosis, previously known as nonspecific vaginitis, *Haemophilus vaginalis* vaginitis, *Corynebacterium vaginale* vaginitis, *Gardnerella vaginalis* vaginitis and anaerobic vaginosis, is an abnormal condition of the vaginal ecosystem caused by overgrowth of both aerobic and anaerobic vaginal bacteria flora[1–4]. It is the most common vaginal disorder in women of reproductive age and is responsible for approximately one-third of all cases of vulvovaginitis. It is now regarded as a risk factor for complications of pregnancy, including chorioamnionitis and prematurity[5–9].

Bacterial vaginosis represents a synergistic polymicrobial infection, characterized by an overgrowth of bacterial species usually found in the vagina. The lactobacilli-dominated flora is replaced by a mixed flora, consisting of *Gardnerella vaginalis*; anaerobes such as *Bacteroides* spp., *Prevotella* spp. and *Mobiluncus* spp.; and *Mycoplasma hominis*[10].

Several bacterial enzymes, including sialidases, have been implicated as virulence factors in pregnancy complications such as prematurity and chorioamnionitis. Sialidases, formerly known

Correspondence to: Jorgelina Smayevsky, PhD, Camargo 581, Piso 4, Buenos Aires 1414, Argentina. E-mail: JSmayevsky@cemic.edu.ar as neurominidases, are enzymes that cleave α-ketosidic linkages between the glycosyl residues of glycoproteins, glycolipids and sialic acids[11]. The purpose of this study was to determine the prevalence of *Gardnerella vaginalis*, anaerobic bacteria and *Mycoplasma hominis* in vaginal specimens from women with and without bacterial vaginosis, as well as to determine the sensitivity and specificity of the direct sialidase assay on vaginal fluid as a rapid test for diagnosing this syndrome.

### MATERIALS AND METHODS

In total 109 women (mean age 33 ± 7.1 years) were studied. Forty-seven women with BV were included in the BV group. Sixty-two women from the same hospital, free of BV, were included as controls. All women were nonpregnant, and none was menstruating at the time of examination. Women were excluded from the study when they had used antibiotics 3 days before the study.

BV was defined by the presence of vaginal pH > 4.5, fishy odor-positive in presence of 10% KOH (positive whiff test), and presence of clue cells. Controls (women without BV) were defined by the absence of all these three clinical criteria. Homogeneous vaginal discharge was not considered. Specimens were taken from the posterior vaginal fornix using a sterile, nonlubricated speculum. Odor was tested by immersing a swab containing the vaginal fluid in 10% KOH and smelling the odor. The pH of vaginal secretions was measured with pH paper (Spezialindikator pH 4.0–7.0; Merck). A second vaginal swab was collected in saline solution and examined microscopically for bacterial morphologic types, clue cells, white cells, trichomonads and yeasts by wet mount (× 400).

Gram stain was performed using a third swab and evaluated for bacterial morphologic types using Nugent's score (× 1000)[12]. Women were categorized as BV-positive (scores 7–10) and BV-negative (score < 7) using the Gram stain scoring system for vaginal smears. Having more than 10 polymorphonuclear cells per field (× 1000) was considered as a significant inflammatory reaction.

Three other vaginal swabs were placed into Cary Blair transport medium, into anaerobic transport medium, and into mycoplasma transport medium. Another vaginal swab was collected to perform a sialidase activity assay. Endocervical swab specimens were taken for screening for *Neisseria gonorrhoeae* and *Chlamydia trachomatis*.

Vaginal swabs were placed onto chocolate agar, human blood agar, modified Thayer-Martin medium and Feimberg medium, as described elsewhere[13]. *G. vaginalis* cultures recovered from the third and fourth streak zones on an agar plate were considered significant.

Brucella blood agar + vitamin K (1 μg/ml) + hemin (5 μg/ml) and brucella blood agar + K vitamin (1 μg/ml) + hemin (5 μg/ml) + amikacin (50 μg/ml) were incubated for 7 days at 35°C in an anaerobic chamber[14]. All other plates were incubated at 37°C in 5% $CO_2$ for 96 h. Feimberg medium was incubated in air for 96 h. *Mobiluncus* culture was not performed.

*Ureaplasma urealyticum* and *Mycoplasma hominis* were cultivated in urea broth, arginine broth and A7 Sheppard agar and incubated for 5 days at 35°C in 5% $CO_2$ atmosphere. *U. urealyticum* cultures were considered as significant when the colony count was higher than $10^4$ ccu/ml[15].

Aerobic and facultative anaerobic isolates were identified by conventional methods[13,16]. Anaerobic bacteria growing in the third and fourth streaks were identified by performing biochemical tests[14,17]. Sialidase activity of anaerobic bacteria was measured by a filter-paper spot test[18]. Selective medium for lactobacilli was not used.

Specimens in 2SP medium were stored frozen at −70°C for up to 5 days. Aliquots of 250 μl were inoculated onto McCoy cells monolayers growing on glass coverslips in shell vials by standard methods[19]. The tissue cultures were incubated at 35°C in a 5% $CO_2$ atmosphere. After 72 h the cultures were stained with Jones's iodine stain and examined microscopically for chlamydial inclusions.

Sialidase activity was qualitatively determined by a filter-paper spot test using a stock solution of 2′-(4-methylumbelliferyl)α-D-N-acetyl-neuraminic acid in buffer acetate. Prior to use, it was diluted and filter paper strips were saturated with this solution. Paper strips were then inoculated with a spot of vaginal fluid and incubated for 15 min at 37°C. The test was interpreted by

**Table 1** Prevalance of microorganisms in the vaginal fluid of 109 women with and without bacterial vaginosis (BV)

| | *BV+ (n = 47)* | | *BV– (n = 62)* | | |
|---|---|---|---|---|---|
| *Microorganisms isolated** | *Number* | *Percentage* | *Number* | *Percentage* | p *value*** |
| Anaerobic Gram-negative rods | | | | | |
| *Prevotella bivia* | 22 | 47 | 1 | 1 | <0.001 |
| *P. intermedia* | 3 | 6 | 1 | 1 | NS |
| *P. disiens* | 1 | 2 | 0 | 0 | NS |
| *Prevotella* spp. | 5 | 10 | 0 | 0 | NS |
| *Porphyromonas asacharolytica* | 17 | 36 | 1 | 1 | <0.05 |
| *Porphyromonas* spp. | 3 | 6 | 1 | 1 | NS |
| *Bacteroides* spp. | 3 | 6 | 0 | 0 | NS |
| Anaerobic Gram-positive rods | | | | | |
| *Mobiluncus* morphotypes | 16 | 34 | 0 | 0 | <0.01 |
| Anaerobic Gram-positive cocci | | | | | |
| *Peptostreptococcus* spp. | 23 | 49 | 8 | 13 | <0.001 |
| Facultative anaerobic bacteria | | | | | |
| *Gardneralla vaginalis* | 36 | 76 | 1 | 1 | <0.001 |
| *Streptococcus agalactiae* | 2 | 4 | 5 | 8 | NS |
| Mycoplasmas | | | | | |
| *Mycoplasma hominis* | 20 | 42 | 0 | 0 | <0.001 |
| *Ureaplasma urealyticum* | 10 | 21 | 9 | 14 | <0.05 |
| Yeasts | | | | | |
| *Candida albicans* | 2 | 4 | 7 | 11 | NS |

**T. vaginalis*, *N. gonorrhoeae* and *C. trachomatis* have not been isolated; **the $\chi^2$ tests and the Fisher's exact test were used examining the strips under a long-wavelength (365 nm) lamp. A fluorescent blue spot was indicative of sialidase activity[11].

The $\chi^2$ tests, Fisher's exact test and Mann–Whitney test were used for comparative analysis of the data obtained, and significance was assigned at $p < 0.05$.

**Table 2** Number of isolates in vaginal samples of 109 women with and without bacterial vaginosis *(BV)*

| *Isolates* | *Women with BV (n = 47)* | *Women without BV (n = 62)* | p *value** |
|---|---|---|---|
| Total number | 163 | 34 | |
| Mean ± SD per specimen | 3.47 ± 1.20 | 0.55 ± 0.71 | <0.001 |

Values are reported as mean ± standard deviation (SD); *the Mann–Whitney test was used

### RESULTS

The prevalence of microorganisms isolated in the vaginal fluid in women with and without BV is shown in Table 1. Neither *Neisseria gonorrhoeae* nor *Chlamydia trachomatis* was isolated. Anaerobic bacteria were isolated in 91% and 18% of the women with and without BV, respectively ($p < 0.001$).

The mean number of isolates per specimen from women with BV was ~4–7 times more than that from those without BV (Table 2). *Peptostreptococcus* spp., *P. bivia, and Porphyromonas* spp. were strongly associated with BV ($p < 0.001$). *P. bivia* and *Prevotella* spp. represented 44% of all of the anaerobes isolated in the BV+ group.

*G. vaginalis* and *M. hominis* were isolated in 76% and 42% of the women with BV and in 1.0% and 0% of the women without BV, respectively ($p < 0.001$). These data showed a strong association between each microorganism and BV.

*Mobiluncus* morphotypes were observed only in 34% of the BV+ group.

All of 33 *P. bivia* strains as well as one of the five isolates of *Prevotella* spp. and one of one isolate of *P. disiens* presented sialidase activity. Vaginal sialidase activity was detected in 81% of the women with BV but in only 6% in the control group ($p < 0.001$). Overall, positive predictive value (PPV) and negative predictive value (NPV) of the vaginal sialidase test for BV diagnosis were 90% and 86%, respectively. Sialidase-positive bacteria were recovered from 51% of the women with BV and from only one patient (1%) among those without BV.

By the Gram stain scoring system for vaginal smear, women with BV were categorized as follows: 2% had intermediate vaginal flora (scores 4–6) and 98% had BV vaginal flora (score ≥ 7). No women with BV had normal vaginal flora (scores 0–3). Ninety-two per cent of the women without BV had normal vaginal flora (scores 0–3), 6% had intermediate vaginal flora and 2% had BV vaginal flora. The PPV and NPV of the Gram stain scoring system were 98% and 98%, respectively.

We did not find any statistical difference between the inflammatory reaction observed in the vaginal Gram stains of the two groups. Four per cent of the patients of the BV+ group and 11% of the patients of the BV− group presented significant inflammatory reaction (PPV 22%, NPV 55%).

### DISCUSSION

To our knowledge this is the first microbiology study of the vaginal microflora of nonpregnant women with and without BV in our country, with special emphasis on anaerobic bacteria. We defined BV by the presence of vaginal pH > 4.5, fishy odor positivity in the presence of 10% KOH (positive whiff test), and presence of clue cells. We decided not to consider homogeneous vaginal discharge because of its low PPV[20]. Thomason *et al.*[20] speculated that this poor predictive value may be attributable to interexaminer variability of this criterion. In the same study, the authors concluded that homogeneous discharge was of little diagnostic value.

Nevertheless, we did not use quantitative methodologies, similar to those used by Hillier *et al.*[21]; we isolated *Prevotella* species, *Peptostreptococcus* spp., and *P. asacharolytica* in most women with BV. These results agree with those published by Puapermpoonsiri and colleagues[22]. who isolated *Prevotella* species (mainly *P. bivia*), *Porphyromonas* species and *Peptostreptococcus* species significantly associated with BV in pregnant Japanese and Thai women. These authors also found that the mean number of organisms recovered in the BV+ group is twice as high as that in the control group[22].

We found a very good sensitivity and specificity of the sialidase activity in the vaginal fluid. Briselden *et al.*[11] described sialidase activity in 84% of women with BV and in none of 19 women with normal vaginal flora. Furthermore, vaginal sialidase was eradicated in 95% of the women after successful treatment but in none of the women with persistent or recurrent BV[11]. Smayevsky *et al.*[23] found also a very good sensitivity and a specificity of a vaginal sialidase assay in 316 nonpregnant women (92% and 94%, respectively).

*Prevotella* species were the only anaerobic bacteria displaying sialidase activity. These data suggest that the presence of sialidase activity in the vaginal fluid of women with BV is mainly associated with the presence of sialidase-positive *P. bivia*. Briselden *et al.*[11] found that all of the 83 *P. bivia* isolates studied were positive for sialidase activity, compared to 12 (38%) of 32 *P. disiens* isolates.

The production of sialidase by the anaerobes associated with prematurity suggests that the enzyme may be involved. Studies have demonstrated that tissue exposure to sialidases eliminates the subterminal sugars, resulting in an increased adherence capability and invasion and destruction of mucosal tissue[3,24]. The presence of *P. bivia* in vaginal fluid has been recently correlated with an important increase in premature birth[4,7,8,25].

We detected *Mobiluncus* morphotypes in 34% of the women with BV and in none of the 62 women without BV. These results agree with those of Spiegel *et al.*[26] and Puapermpoonsiri *et al.*[22], but these authors did perform cultures for detecting *Mobiluncus* species.

We isolated *M. hominis* in 42% of the women with BV. Taylor-Robinson[27] isolated *M. hominis* in 60% of the women with BV and in 10% of the women without BV, and Smayevsky *et al.*[28] described, for women with BV, a strong association between *M. hominis* and *G. vaginalis* (82%). These data emphasize the close correlation between *M. hominis* and BV.

It has already been noted that *G. vaginalis* can be recovered from vaginal fluid of normal women[1,2]. Several authors have described *G. vaginalis* isolation rates ranging from 36% to 55% in women without BV[2,3,6]. In contrast, we found *G. vaginalis* in only 1% of the women without BV. This difference may be explained on the basis of inclusion criteria and methodology differences. First, we defined our negative control group (women without BV) as patients in whom none of the three clinical signs – pH, whiff test, and clue cells – was present, whereas women who did not meet three of the four Amsel criteria were included in the non-BV group by others. On the other hand, we considered significant *G. vaginalis* growth, recovered from third and fourth streaks.

Our study is in agreement with previous data showing that the presence of a significant inflammatory reaction is neither sensitive nor specific for diagnosing BV[20], because BV is considered to be a noninflammatory disease[2]. We conclude that anaerobic bacteria, especially *P. bivia, G. vaginalis* and *M. hominis*, are the organisms most involved in BV. The sialidase activity in vaginal fluid and the Gram stain scoring system represent accurate, rapid and inexpensive methods for detection of bacterial vaginosis.

### REFERENCES

1. Isson C, Taylor-Robinson D. Bacterial vaginosis. *Int J STD AIDS* 1997;8:2–3
2. Eschenbach D, Hillier SL, Crichlow C, *et al.* Diagnosis and clinical manifestations of bacterial vaginosis. *Am J Obstet Gynecol* 1988;158:819–28
3. Amsel R, Totten PA, Spiegel CA, *et al.* Non-specific vaginitis: diagnostic criteria and microbial and epidemiologic associations. *Am J Med* 1983; 74:14–22
4. Holst EB, Wathne B, Hovelius B, *et al.* Bacterial vaginosis: microbiological and clinical findings. *Eur J Clin Microbiol* 1967;6:536–41
5. Hillier SL, Maritus J, Krohn M, *et al.* A case control study of chorioamnionic infection and histologic chorioamnionitis in prematury. *N Engl J Med* 1988; 319:972–8
6. Hillier SL. Diagnostic microbiology of bacterial vaginosis. *Am J Obstet Gynecol* 1993;169:455–9
7. Gravett M, Hummel D, Eschenbach D, Holmes KK. Preterm labor associated with subclinical amniotic fluid infection and with bacterial vaginosis. *Obstet Gynecol* 1986;67:229–37
8. Clark P, Kurtzer P, Duff P. Role of bacterial vaginosis in peripartum infections. *Infect Dis Obstet Gynecol* 1994;2:179–83
9. Holst EB, Goffeng A, Andersch B. Bacterial vaginosis and vaginal microorganisms in idiopathic premature labor and association with pregnancy outcome. *J Clin Microbiol* 1994;32:176–86
10. Spiegel CA. Bacterial vaginosis: changes in laboratory practice. *Clin Microbiol Newslett* 1999; 21:33–7
11. Briselden A, Moncla B, Stevens C, Hillier SL. Sialidases in bacterial vaginosis and bacterial vaginosis-associated microflora. *J Clin Microbiol* 1992;30:663–6
12. Nugent R, Krohn M, Hillier SL. Reliability of diagnosing bacterial vaginosis is improved by a standardized method of Gram stain interpretation. *J Clin Microbiol* 1991;29:297–301
13. Baron EJ, Cassel G, Duffy L, *et al. Cumitech 17 A: Laboratory Diagnosis of Female Genital Tract Infections.* Washington, DC: American Society for Microbiology, 1993
14. Summanen P, Baron EJ, Citron DM, *et al. Wadsworth Anaerobic Bacteriology Manual*, 5th edn. Belmont, CA: Star Publishing Company, 1993
15. Shepard MC. Culture media for ureaplasmas. In Razin S, Tully JG, eds. *Methods in Mycoplasmology,* Vol I. New York: Academic Press, 1983:397–401
16. Funke G, Bernard K. Corynebacterium Gram-positive rods. In Murray P, ed. *Manual of Clinical Microbiology.* Washington, DC: American Society for Microbiology, 1999:319–45
17. Moncla BJ, Braham P, Rabe L, Hillier SL. Rapid presumptive identification of black-pigmented gram negative anaerobic bacteria by using

4-methylumbelliferone derivates. *J Clin Microbiol* 1991;29:1955–8
18. Moncla BJ, Braham P, Hillier SL. Sialidase (neuraminidase) activity among gram-negative anaerobic and capnophilic bacteria. *J Clin Microbiol* 1990;28:422–5
19. Wallace C, Kenny G, Schachter J. *Cumitech 19: Laboratory Diagnosis of Chlamydial and Mycoplasmal Infections.* Washington, DC: American Society for Microbiology, 1984
20. Thomason JL, Gelbart S, Anderson RJ, *et al.* Statistical evaluation of diagonostic criteria for bacterial vaginosis. *Am J Obstet Gynecol* 1990;162: 155–60
21. Hillier SL, Krohn MA, Rabe LK, *et al.* The normal vaginal flora, $H_2O_2$ producing lactobacilli and bacterial vaginosis in pregnant women. *Clin Infect Dis* 1993;16(Suppl):S273–S281
22. Puapermpoonsiri S, Kato N, Watanabe K, *et al.* Vaginal microflora associated with bacterial vaginosis in Japanese and Thai pregnant women. *Clin Infect Dis* 1996;23:748–52
23. Smayevsky J, Roldan L, Fernandez Canigia L, *et al.* Utility of the Gram stain and the sialidase detection for diagnosis of bacterial vaginosis in non-pregnant women. *Int J Gynecol Obstet* 1999;67(Suppl):S51
24. Odin L. Sialic acid in human cervical mucus, in hog seminal gel and in ovomucin. *Acta Chem Scand* 1955;9:1235–6
25. Catlin BW. *Gardnerella vaginalis*: characteristics, clinical considerations and controversies. *Clin Microbiol Rev* 1992;5:213–37
26. Spiegel C, Eschenbach D, Amsel R, Holmes KK. Curved anaerobic bacteria in bacterial non specified vaginosis and their response to anti-microbial therapy. *J Infect Dis* 1983;148:817–23
27. Taylor-Robinson D. Infections due to species of mycoplasmas and ureaplasma: un update. *Clin Infect Dis* 1996;23:671–82
28. Smayevsky J, Bianchini H, Bagnati E, *et al.* Aislamiento de *Gardnerella vaginalis* y *Mycoplasma hominis* en vaginosis bacteriana. *Infect Microbiol Clin* 1989;1:59–61

Received 7/12/00; Accepted 11/10/00
